# Mechanotransduction in Prokaryotes: A Possible Mechanism of Spaceflight Adaptation

**DOI:** 10.3390/life11010033

**Published:** 2021-01-07

**Authors:** Patricia Fajardo-Cavazos, Wayne L. Nicholson

**Affiliations:** Space Life Sciences Laboratory, Department of Microbiology and Cell Science, University of Florida, 505 Odyssey Way, Merritt Island, FL 32953, USA; pfajardo@ufl.edu

**Keywords:** bacteria, gravity, mechanosensing, mechanotransduction

## Abstract

Our understanding of the mechanisms of microgravity perception and response in prokaryotes (Bacteria and Archaea) lag behind those which have been elucidated in eukaryotic organisms. In this hypothesis paper, we: (i) review how eukaryotic cells sense and respond to microgravity using various pathways responsive to unloading of mechanical stress; (ii) we observe that prokaryotic cells possess many structures analogous to mechanosensitive structures in eukaryotes; (iii) we review current evidence indicating that prokaryotes also possess active mechanosensing and mechanotransduction mechanisms; and (iv) we propose a complete mechanotransduction model including mechanisms by which mechanical signals may be transduced to the gene expression apparatus through alterations in bacterial nucleoid architecture, DNA supercoiling, and epigenetic pathways.

## 1. Introduction

On 17 December 1946, the first documented experiment to study the response of a living organism to spaceflight was performed, with the launch and recovery of fungal spores on a V-2 rocket from White Sands, New Mexico [1]. Since that time, considerable theoretical and experimental effort has been devoted to understanding how living organisms sense, respond, and adapt to long-term spaceflight, particularly regarding their exposure to chronic microgravity. Extensive investigations conducted in space have resulted in a relative wealth of understanding of microgravity effects at the mechanistic level in eukaryotic organisms including humans [2], animals [3], plants [4], and protists [5]. In contrast to the situation in eukaryotes, it has proven more difficult to elucidate at the mechanistic level how single-celled prokaryotes (Bacteria and Archaea) sense and respond to reduced gravity [6,7,8].

In this hypothesis paper, we briefly review the state of our understanding of how eukaryotic cells sense, respond, and adapt to spaceflight microgravity, via various pathways responsive to the unloading of mechanical stress. We observe that prokaryotic cells possess many structures analogous to mechanosensitive structures in eukaryotes. We review and update the evidence indicating that prokaryotes possess and actively utilize mechanosensing and mechanotransduction mechanisms, and we expand upon existing models to include the role of the bacterial nucleoid in converting external mechanical information into the modulation of gene expression. This hypothesis builds upon, updates, and expands previous work in the field, to which the reader is referred for background information [9,10].

## 2. Mechanosensing in Eukaryotes

**Figure 1:**
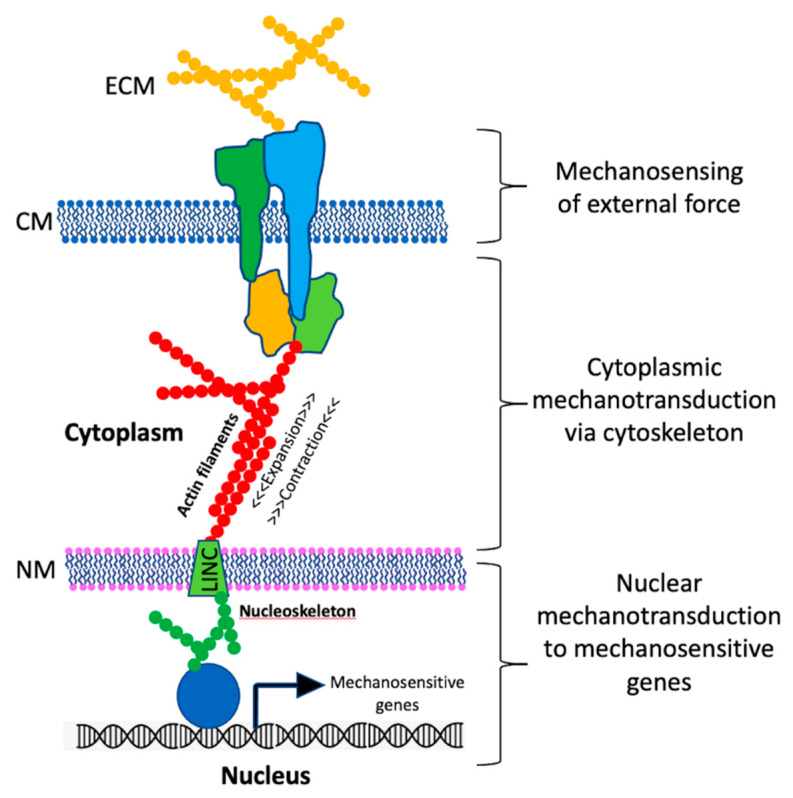
Schematic model of eukaryotic mechanotransduction pathway. An external mechanical force generated through the extracellular matrix (ECM) or tight junctions is sensed by mechanosensitive proteins in the cytoplasmic membrane (CM) and transduced through the cytoskeleton, LINC protein complexes in the nuclear membrane (NM), and nucleoskeleton to effect transcription of mechanosensitive genes. Colored shapes represent proteins in the transduction pathway; their specific identities and functions are elaborated in refs. [15,16,17]. See text for details.

Terrestrial life on Earth has evolved in the continuous presence of gravity [10], and eukaryotic organisms have developed systems to sense their orientation in the gravity field and to respond appropriately. A large body of evidence has accumulated supporting the idea that mechanics play a key role in regulating various eukaryotic cell functions such as cell division, differentiation, immunity, development, and motility. Many of these diverse cellular functions are mediated through pathways of mechanosensing and mechanotransduction, the molecular details of which are currently being elucidated [11,12,13,14]. A generalized and simplified schematic diagram of the steps involved in eukaryotic mechanotransduction is presented in Figure 1 and described below.

In the simplified model depicted in Figure 1, external force can consist of any number of mechanical phenomena, such as shear, stretch, compression, or sedimentation of otoliths or statoliths. Physical forces can also be exerted on a cell through interaction with the extracellular matrix, or with other cells connected via tight junctions. In addition, while not strictly an “external” force, because the nucleus itself is suspended in the cytoskeletal matrix, and its density differs from the surrounding cytoplasm, the nucleus itself exerts mechanical stress on the cell.

The simplified model above depicts an idealized mechanoreceptor as a protein complex embedded in the cytoplasmic membrane that senses external physical forces (Figure 1). The actual situation is almost certainly more complex, and likely differs considerably depending on the type of receptor, cell, tissue, or organism studied. On the simpler end of the spectrum are single transmembrane mechanosensitive ion channels that respond to membrane deformation [18]. On a more complex level, the surfaces of many eukaryotic cells exhibit focal adhesion (FA) structures which physically link the extracellular matrix to the internal cytoskeleton through complexes of signaling proteins, among them mechanosensitive transmembrane proteins. Such structures establish both physical and functional connections between the cell and its external environment (reviewed in [19,20]).

As DNA is separated from the cytoplasmic compartment and contained within a membrane-bound nucleus (Figure 1), how do mechanical signals reach the DNA to modulate gene expression? In addition to the cytoplasmic cytoskeleton, the nucleus itself contains a skeleton (the nucleoskeleton) composed of actin and lamin filaments. Furthermore, the cytoskeleton and nucleoskeleton are physically connected through Linker of Nucleus and Cytoskeleton (LINC) protein complexes [16] (Figure 1). Evidence is accumulating that LINC complexes allow for the rapid mechanotransduction of signals from the cytoplasmic membrane and cytoskeleton directly through the nuclear membrane to the nucleoskeleton, enabling rapid gene expression responses (reviewed in [17]). It is thought that the LINC complex is intimately involved in modulation of genome expression in response to mechanical stimuli through chromatin remodeling and recruitment of DNA and RNA polymerases, ligases, acetylases, methylases, and cyclin-dependent kinases, resulting in changes in gene expression at the transcriptional, translational, post-translational, and epigenetic levels [19,20]. The details of the myriad of eukaryotic mechanotransduction systems, though not yet completely elucidated, are currently being actively investigated [17].

In the freefall environment of spaceflight, gravitational loading is negated. Numerous studies using various cell types have shown that eukaryotic cells exhibit a morphological sensitivity to microgravity, exposure to which can cause alterations in cell shape, size, volume, and adherence properties [10,19,20]. These signals are converted into alterations in chromosomal architecture, which in turn regulate the transcriptional apparatus [21]. In spaceflight research, this general “mechanotransduction” model successfully explains the pathways for sensing and response to microgravity by plant and mammalian whole organisms, as well as single eukaryotic cells [5,22,23]. It is unclear whether such responses to microgravity are direct or indirect (resulting from, say, downstream effects of microgravity on chemical gradients or fluid shear). However, results from parabolic aircraft and sounding rocket flights demonstrated that the transcriptomes of human monocytes responded immediately—within seconds—after exposure to microgravity or hypergravity (1.8 × *g*) [24] suggesting that, in this case at least, gravity or its absence is exerting a direct effect on gene expression.

## 3. Does a Microbial Analog of Eukaryotic Mechanotransduction Exist? 

In contrast to our relatively detailed understanding of the eukaryotic microgravity response in terms of mechanosensing and mechanotransduction mechanisms, no such detailed model exists to explain how prokaryotes respond to gravity in general, much less to spaceflight microgravity. The origins of this situation can be traced back to an influential and often-cited theoretical study from 1965 asserting that microorganisms smaller than 10 micrometers should not be able to perceive gravitational forces [25]. Despite a lack of empirical evidence supporting this contention, it has nevertheless persisted in the spaceflight microgravity literature over the ensuing half century (for reviews, see [8,26]). 

Due to a lack of fluid convection in microgravity, spaceflight has been considered to be a quiescent environment characterized by low hydrodynamic shear and lack of convective transfer of mass and heat [26]. To emulate this low-shear environment on Earth, various rotating devices called clinostats have been developed and utilized as microgravity analog systems [27,28]. Early experiments cultivating microbes in clinostats indicated that certain phenotypes such as microcin B17 production by *Escherichia coli* [29] and virulence in *Salmonella enterica* [30] were altered in the clinostat environment. These and similar observations led to the notion of mechanotransduction as a possible mechanism for a microgravity response in prokaryotes, proposed in a 2004 review [9]. Numerous subsequent investigations have been conducted to characterize the response of various microorganisms to the low fluid shear environment using both spaceflight and clinorotation ([31,32,33,34,35]; reviewed extensively in [7,8,26]). These studies clearly established a role for hydrodynamic shear stress, or its absence, in influencing some aspects of gene expression and some phenotypes in some, but not all, bacteria tested [7,8]. Subsequent work mapping transcriptomic responses of *S. enterica* and *Pseudomonas aeruginosa* to spaceflight and clinorotation identified the molecular chaperone and global regulator Hfq as being implicated in the low-shear response, although its mechanism of action is currently unknown [33,34,36]. Several studies have mapped global transcriptomic responses of bacteria to the low-shear environment, comparing cells grown in spaceflight and clinostat conditions with corresponding ground controls [33,34,36,37,38,39], but to date, these studies have failed to yield insights into a detailed underlying molecular mechanism [40,41].

### 3.1. Microbial Internal Structure

If we are to draw upon the eukaryotic mechanotransduction model of spaceflight signal transduction as depicted in Figure 1 and extend it to microbes, it is worthwhile first to review our current understanding of the internal structure of prokaryotes, paying particular attention to structures involved in mechanosensing and mechanotransduction. Our understanding of the interior architecture of living microbial cells has lagged behind that of eukaryotes, due to their small size at the limits of optical resolution and the lack of techniques for mechanical manipulation of such small cells [42,43]. Indeed, until relatively recently the interior of the prokaryotic cell was thought to be an unordered “bag of enzymes” with little or no intracellular compartmentalization [44]. Advances in molecular biology, microscopic imaging technologies, and the development of fluorescent molecular tags have changed this view radically within the past two decades; microbial cells are now known to exhibit a high level of subcellular organization rivaling that of eukaryotic cells [45,46,47].

As was mentioned in a previous review [9], microbes possess both functional and structural analogs of the eukaryotic cytoskeleton; indeed, cytoskeletal components from all three domains of life (Bacteria, Archaea, and Eukarya) are phylogenetically related, indicating their descent from a common ancestor early in evolution [48]. An updated comparison of eukaryotic and prokaryotic cytoskeletal analogs is presented in Table 1.

As can be seen in Table 1, numerous proteins belonging to both the actin and tubulin superfamilies of eukaryotic cytoskeletal proteins have been identified in Bacteria and Archaea. In addition to the canonical actin/tubulin/intermediate filament superfamilies, prokaryotes contain a large number of filament-forming proteins that do not have eukaryotic homologs, such as ParA/B, MinC/D, and bactofilins [49]. Many of these protein filaments organize collaboratively through interactions between themselves and supramacromolecular structures such as the bacterial membrane, cell wall, and nucleoid [49]. In prokaryotic cells, internal filaments serve a variety of functions, including: organizing cell division; control of cell shape; organization and segregation of chromosomal, plasmid, and phage DNA; membrane remodeling; organization of cell wall synthesis; and scaffolding of spore structures in spore-forming Firmicutes [45,46,47,50]. In addition, in many cases cytoskeletal filaments have been directly visualized and characterized in prokaryotic cells using high-resolution fluorescence microscopy and cryo-electron microscopy techniques [50,51].

### 3.2. Mechanosensing in Prokaryotes

Since the early days of microbiology in the 19th century, prokaryotes have been known to monitor and respond to their external chemical environment [52]. More recently, microbes have also been shown to respond to mechanical forces such as fluid flow, pressure, and contact with surfaces or other cells [9,45,46]. Similar to eukaryotic cells, bacteria possess mechanosensory proteins either displayed on the cell surface (e.g., adhesins) or extending into the extracellular environment (e.g., flagella, pili). Proteins residing in the cell envelope can sense and respond to deformations of the cell surface. The integral membrane proteins MscS and MscL sense membrane deformation resulting from osmotic stress and respond by opening or closing their pores to allow passage of osmotically active solutes (e.g., potassium, glutamate, or trehalose) in order to maintain osmotic balance and turgor pressure [53]. Adhesins present on the cell surface are able to sense forces such as shear, compression, or tension and transduce these signals to the cell interior, activating the production of second messenger molecules such as cAMP and cyclic di-GMP which in turn activate or repress transcription of target genes [45]. Protein filaments such as flagella and Type IV pili are anchored in the cell surface and extend into the extracellular environment. These structures have long been associated with motility (flagella) and adhesion (pili), but recently have also been implicated in the regulation of gene expression via mechanosensing and mechanotransduction mechanisms (reviewed in [46]). For example, motility of Gram-positive *Bacillus subtilis* cells through liquid media is due to the rotation of helical flagella driven by rotary motors embedded in the cell membrane. Contact of cells with a surface increases the torque applied onto flagella, inhibiting their rotation. This rotational inhibition increases strain on the flagellar motor embedded in the membrane, which generates a signal sensed by the two-component system histidine kinase DegS. DegS in turn phosphorylates the DegU response regulator, and phosphorylated DegU up-regulates the transcription of an entire class of genes involved in biofilm production [54,55]. Thus, an outside mechanical force can be sensed by a bacterial cell and transduced to the transcriptional apparatus to dramatically change its phenotype from a solitary free-swimming cell to a sessile member of a biofilm community. 

### 3.3. Mechanotransduction in Prokaryotes

In the above examples, perception of mechanical stress was shown to trigger classic chemical transducers, such as second messengers or two-component systems, to effect altered gene expression. Such mechanisms do not rely on mechanotransduction through a cytoskeleton. Indeed, at present cytoskeletal mechanotransduction in prokaryotes has been much less thoroughly investigated than its eukaryotic counterpart, although recent studies have begun to uncover the roles played by prokaryotic cytoskeletal protein homologs in the regulation of cell physiology. Some examples follow.

#### 3.3.1. Cell Growth and Division

The prokaryotic cell wall is a dynamic network of peptidoglycan (PG) making up the sacculus that maintains cell shape, resists lysis of the cell by turgor pressure, and which must accommodate cell growth in varying environments. Morphogenesis of the cell wall is ultimately governed by cytoskeletal proteins (reviewed recently in [56]). At least two distinct machineries exist to accomplish this feat, the elongasome and the divisome. Filaments of MreB, FtsZ, and FtsA proteins serve to organize and direct the growth and division machinery to their proper locations. 

The elongasome is responsible for the insertion of new PG into the growing sacculus and is organized by the actin homolog MreB (Table 1). MreB filaments move circumferentially around rod-shaped cells and organize the multiprotein PG synthetic machinery to add PG in a helical fashion along the cell cylinder, allowing cell length to increase [49]. 

The divisome is the molecular machine responsible for cell separation (cytokinesis) under the control of the tubulin homolog FtsZ and the actin homolog FtsA (Table 1). FtsZ filaments organize into a centrally located ring-shaped structure (the Z-ring) that directs the PG machinery to produce the division septum during cytokinesis [56]. Cytokinesis also involves the separation of the cell membrane. FtsA forms filaments that directly interact with FtsZ filaments and anchor the constricting Z-ring to the cytoplasmic membrane, ensuring that all layers of the cell envelope are constricted and separated during cytokinesis [49]. In addition, placement of the division septum at mid-cell and suppression of division at the cell poles is accomplished by filaments of DivIVA, MinC, and MinD which form a complex structure at the cell poles [49]. 

#### 3.3.2. DNA Division, Repair, and Gene Expression

In prokaryotes, filament-forming proteins perform numerous functions on DNA including chromosome division, chromosome and plasmid segregation, DNA repair, and regulation of gene expression (reviewed in [49]). The actin homolog MreB is involved with the initial stages of bacterial chromosome segregation coupled with DNA synthesis [57]. With help of ParB, the ParA protein forms collaborative filaments with DNA and is involved with chromosome movement and segregation during cell division using a currently unknown mechanism [49]. The RecA protein is central both in DNA repair and homologous recombination; it binds and forms filaments with both single- and double-stranded DNA and has been shown to exert cytomotive properties in the movement of DNA during double-strand break repair [49]. H-NS proteins are a class of transcriptional repressors that nucleate at specific regulatory sequences and form stiff collaborative filaments that sterically hinder RNA polymerase from binding to promoters in the vicinity. It is estimated H-NS regulates the transcription of up to 5% of the genes in *E. coli* [49]. However, at present, the mechanism(s) of exactly how cytoskeletal filaments are involved in coordinating DNA synthesis and chromosomal division with membrane and cell wall synthesis and cytokinesis in prokaryotes is currently unknown and an active area of research.

### 3.4. Prokaryotic Nucleoid Architecture

The prokaryotic counterpart of the eukaryotic nucleus is the bacterial chromosome or nucleoid. The nucleoid consists of genomic DNA in a complex with its complement of nucleoid-associated proteins (NAPs). The nucleoid is not bounded by its own membrane and is in direct contact with the cytoplasm. In the last two decades, using fluorescence microscopy to track chromosomal loci inside bacteria cells, researchers have found that bacterial chromosomes are organized into stereotypical patterns, with specific chromosomal regions localizing to defined cellular locations (reviewed in [58]). Over the past few years, the application of genome-wide high-resolution chromosome conformation capture (Hi-C) assays to bacteria has provided a detailed picture of the three-dimensional folding characteristics of the entire chromosome. In parallel, super-resolution microscopy techniques have strongly advanced our knowledge of bacterial nucleoid organization. 

It is increasingly appreciated that the nucleoid adopts a specific architecture, which alters dramatically in response to the growth phase, cellular development, and environmental changes. Recent Hi-C studies of a number of bacterial species provided insights into the three-dimensional folding of the bacterial chromosome. In *Caulobacter crescentus* and *B. subtilis*, but not *E. coli*, the chromosome exhibits long-range interactions. The two replication arms are juxtaposed from the origin to the terminus region, mediated by the SMC condensin complex [59,60,61,62,63]. For all three bacterial species, the genome along its entire length is organized into chromosome interaction domains (CIDs), ranging from ~30 to >400 kilobase pairs. Regions within each domain interact with each other more frequently than with regions in other domains. The domain boundaries are often enriched with highly transcribed genes, and inhibition of transcription essentially abolishes all domain boundaries [59,63], indicating that the 3D architecture of the nucleoid is intimately linked to transcription.

### 3.5. DNA Supercoiling

The nucleoid is a highly condensed structure that must allow the replication and transcription machinery to access specific genes in a coordinated fashion, in order to optimize cell metabolism and replication under a wide range of environmental conditions [64]. To accomplish this feat, selected parts of the nucleoid are constantly and dynamically de- and re-condensed in response to environmental signals. Dynamic restructuring of the nucleoid depends critically on a host of enzymes that control DNA topology, collectively known as topoisomerases, which maintain DNA in an optimal state of negative supercoiling. Transcription of a gene is dependent upon the local DNA superhelical state, and in turn, the transcription process itself influences DNA supercoiling, by overwinding DNA ahead of the transcription complex and underwinding it behind (reviewed in [65]). A growing body of evidence points to changes in DNA supercoiling as being a key mediator between environmental sensing by a microbe and its response through modulation of global transcription patterns. It has been shown experimentally that DNA supercoiling is sensitive to variations in the physical environment such as: pH, oxygen, temperature, osmotic pressure, oxidative stress, nutrition, and growth phase, among others (reviewed in [66]). In turn, it has also been shown experimentally that alterations in DNA supercoiling can influence the global pattern of bacterial gene transcription, and ultimately the phenotypic response to environmental changes (reviewed in [66]). Historically, most studies in this area have been performed using Gram-negative bacteria (*E. coli, S. enterica, Vibrio cholerae*); however, these investigations have been recently extended to the Gram-positive opportunist *Streptococcus pneumoniae* [67]. Recent evidence suggests active transcription drives the formation of boundaries between CIDs [68], likely through alterations in DNA topology [65], thus highlighting the three-way interactions between nucleoid architecture, supercoiling, and transcription.

### 3.6. Epigenetic Modification of DNA

Epigenetics is the study of heritable changes in gene expression that occur without changes in DNA sequence (reviewed in [69]). In bacteria, the most-studied epigenetic modification of DNA is base methylation. Specific DNA methyltransferases are responsible for the post-replicative addition of a methyl group to the N6-position of adenine (6mA), the C5-position of cytosine (5mC), or the N4-position of cytosine (4mC) [70]. Base methylation can modulate the interaction of DNA-binding proteins with their cognate sites, and methylation of DNA controls genome defense, chromosome replication and segregation, nucleoid organization, cell cycle control, DNA repair, and regulation of transcription (reviewed in [71]). In particular, a number of specific systems have been described in which gene transcription is directly regulated by DNA methylation/demethylation/hemimethylation ([72,73,74,75,76,77]; reviewed in [71]). Furthermore, both nucleoid organization and chromosome replication are controlled by DNA methylation [78,79]. Therefore, the available evidence indicates that nucleoid architecture, DNA supercoiling, epigenetic DNA modification, and transcription are all tightly linked in prokaryotes.

## 4. Does Mechanotransduction Play a Role in the Prokaryotic Microgravity Response?

From the examination of the studies cited above, we have seen that eukaryotic cells possess mechanosensing and mechanotransduction systems that modulate their gene expression in response to microgravity. Further, we have seen from the results of recent studies utilizing model bacterial species that it is becoming increasingly clear that prokaryotes also possess complex intracellular compartmentalization, analogs of cytoskeletal proteins, and sophisticated signal transduction networks comparable to those of eukaryotic cells. These networks serve to sense external environmental cues through surface structures and to transmit this information to the transcription apparatus via traditional signaling pathways such as two-component systems. From the available information and by analogy, we hypothesize that the prokaryotic cytoskeletal apparatus functions in an analogous manner to its eukaryotic counterpart. We further propose that the microbial cytoskeleton plays a key role in transducing mechanical changes sensed at the cell surface to the nucleoid, altering its architecture and the global transcriptional apparatus. Testing of this hypothesis will necessitate conducting new types of experiments in the spaceflight environment, such as: visualization of cytoskeletal structures, mapping of nucleoid architecture, and measurements of DNA supercoiling and methylation patterns, to name a few. As many of these measurements can be very sensitive to differences in sample handling, it will be of high importance in this endeavor to match as precisely as possible media and growth conditions, spaceflight hardware, and pre- and post-flight sample handling, to eliminate all possible variables with the exception of gravity. Ideally, it would be preferable to perform all exposures in the microgravity environment (providing 1 × *g* by centrifugation) and to perform all inoculations, fixations, freezing, etc. at the same time and using the same protocols. Results from such experiments hold the promise for uncovering a new and fundamental mechanism for microbial adaptation to spaceflight microgravity, mechanosensing and mechanotransduction.

## Figures and Tables

**Table 1:** Comparison of cytoskeletal analogs ^1^.

Eukaryotic Cytoskeletal Structure	Eukaryotic Subunit Analogs	Prokaryotic Subunit Analogs	Function in Prokaryotes
Microtubules	α-Tubulin, β-Tubulin	FtsZ	Cytokinesis
		TubZ	DNA positioning
		RepX	Plasmid replication
Microfilaments	Actin Superfamily	MreB	Cell shape, chromosome segregation
		FtsA	Cytokinesis
		ParM	DNA segregation
Intermediate Filaments	Keratins, Vimentin, Desmin, Neurofilament Proteins, Lamins	CreS (crescentin)	Cell shape

^1^ Adapted from [48].

## References

[1] Demets R., Ouwehand L. (2011). Biology on sounding rockets: History, requirements, results, and scientific interpretation. Proceedings of the 20th Symposium on European Rocket and Balloon Programmes and Related Research.

[2] Pietsch J., Bauer J., Egli M., Infanger M., Wise P., Ulbrich C., Grimm D. (2011). The effects of weightlessness on the human organism and mammalian cells. Curr. Mol. Med..

[3] Lychakov D.V. (2016). Behavioural and functional vestibular disorders after space flight: 2. Fish, amphibians and birds. J. Evol. Biochem. Physiol..

[4] Vandenbrink J.P., Kiss J.Z. (2016). Space, the final frontier: A critical review of recent experiments performed in microgravity. Plant. Sci..

[5] Hader D.P., Braun M., Grimm D., Hemmersbach R. (2017). Gravireceptors in eukaryotes-a comparison of case studies on the cellular level. NPJ Microgravity.

[6] Rosenzweig J.A., Abogunde O., Thomas K., Lawal A., Nguyen Y.U., Sodipe A., Jejelowo O. (2010). Spaceflight and modeled microgravity effects on microbial growth and virulence. Appl. Microbiol. Biotechnol..

[7] Rosenzweig J.A., Ahmed S., Eunson J., Chopra A.K. (2014). Low-shear force associated with modeled microgravity and spaceflight does not similarly impact the virulence of notable bacterial pathogens. Appl. Microbiol. Biotechnol..

[8] Taylor P.W. (2015). Impact of space flight on bacterial virulence and antibiotic susceptibility. Infect. Drug Resist..

[9] Nickerson C.A., Ott C.M., Wilson J.W., Ramamurthy R., Pierson D.L. (2004). Microbial responses to microgravity and other low-shear environments. Microbiol. Mol. Biol. Rev..

[10] Najrana T., Sanchez-Esteban J. (2016). Mechanotransduction as an adaptation to gravity. Front. Pediatr.

[11] DuFort C.C., Paszek M.J., Weaver V.M. (2011). Balancing forces: Architectural control of mechanotransduction. Nat. Rev. Mol. Cell Biology.

[12] Luo T., Mohan K., Iglesias P.A., Robinson D.N. (2013). Molecular mechanisms of cellular mechanosensing. Nat. Mater..

[13] Moulia B., Coutand C., Julien J.L. (2015). Mechanosensitive control of plant growth: Bearing the load, sensing, transducing, and responding. Front. Plant. Sci..

[14] Lindstrom J.B., Pierce N.T., Latz M.I. (2017). Role of trp channels in dinoflagellate mechanotransduction. Biol. Bull..

[15] Martino F., Perestrelo A.R., Vinarský V., Pagliari S., Forte G. (2018). Cellular mechanotransduction: From tension to function. Front. Physiol..

[16] Sankaran J., Uzer G., van Wijnen A.J., Rubin J. (2019). Gene regulation through dynamic actin control of nuclear structure. Exp. Biol. Med..

[17] Piccus R., Brayson D. (2020). The nuclear envelope: Lincing tissue mechanics to genome regulation in cardiac and skeletal muscle. Biol. Lett..

[18] Haswell E.S., Phillips R., Rees D.C. (2011). Mechanosensitive channels: What can they do and how do they do it?. Structure.

[19] Aventaggiato M., Barreca F., Vernucci E., Bizzarri M., Ferretti E., Russo M.A., Tafani M. (2020). Putative receptors for gravity sensing in mammalian cells: The effects of microgravity. Appl. Sci..

[20] Bradbury P., Wu H., Choi J.U., Rowan A.E., Zhang H., Poole K., Lauko J., Chou J. (2020). Modeling the impact of microgravity at the cellular level: Implications for human disease. Front. Cell Dev. Biol..

[21] Uhler C., Shivashankar G.V. (2017). Regulation of genome organization and gene expression by nuclear mechanotransduction. Nat. Rev. Molec. Cell Biol..

[22] Paul A.L., Zupanska A.K., Schultz E.R., Ferl R.J. (2013). Organ-specific remodeling of the *Arabidopsis* transcriptome in response to spaceflight. BMC Plant Biol..

[23] Vorselen D., Roos W.H., MacKintosh F.C., Wuite G.J.L., van Loon J. (2014). The role of the cytoskeleton in sensing changes in gravity by nonspecialized cells. FASEB J..

[24] Thiel C.S., Tauber S., Christoffel S., Huge A., Lauber B.A., Polzer J., Paulsen K., Lier H., Engelmann F., Schmitz B. (2018). Rapid coupling between gravitational forces and the transcriptome in human myelomonocytic U937 cells. Sci. Rep..

[25] Pollard E.C. (1965). Theoretical studies on living systems in the absence of mechanical stress. J. Theor. Biol..

[26] Horneck G., Klaus D.M., Mancinelli R.L. (2010). Space microbiology. Microbiol. Mol. Biol. Rev..

[27] Anken R. (2013). Simulation of microgravity for studies in gravitational biology: Principles, devices, and applications. Curr. Biotechnol..

[28] Herranz R., Anken R., Boonstra J., Braun M., Christianen P.C., de Geest M., Hauslage J., Hilbig R., Hill R.J., Lebert M. (2013). Ground-based facilities for simulation of microgravity: Organism-specific recommendations for their use, and recommended terminology. Astrobiology.

[29] Fang A., Pierson D.L., Koenig D.W., Mishra S.K., Demain A.L. (1997). Effect of simulated microgravity and shear stress on microcin B17 production by *Escherichia coli* and on its excretion into the medium. Appl. Environ. Microbiol..

[30] Nickerson C.A., Ott C.M., Mister S.J., Morrow B.J., Burns-Keliher L., Pierson D.L. (2000). Microgravity as a novel environmental signal affecting *Salmonella enterica* serovar Typhimurium virulence. Infect. Immun..

[31] Crabbé A., Nielsen-Preiss S.M., Woolley C.M., Barrila J., Buchanan K., McCracken J., Inglis D.O., Searles S.C., Nelman-Gonzalez M.A., Ott C.M. (2013). Spaceflight enhances cell aggregation and random budding in *Candida albicans*. PLoS ONE.

[32] Aunins T.R., Erickson K.E., Prasad N., Levy S.E., Jones A., Shrestha S., Mastracchio R., Stodieck L., Klaus D., Zea L. (2018). Spaceflight modifies *Escherichia coli* gene expression in response to antibioticc exposure and reveals role of oxidative stress response. Front. Microbiol..

[33] Crabbé A., Schurr M.J., Monsieurs P., Morici L., Schurr J., Wilson J.W., Ott C.M., Tsaprailis G., Pierson D.L., Stefanyshyn-Piper H. (2011). Transcriptional and proteomic responses of *Pseudomonas aeruginosa* PAO1 to spaceflight conditions involve Hfq regulation and reveal a role for oxygen. Appl. Environ. Microbiol..

[34] Wilson J.W., Ott C.M., Bentrup K.H.Z., Ramamurthy R., Quick L., Porwollik S., Cheng P., McClelland M., Tsaprailis G., Radabaugh T. (2007). Space flight alters bacterial gene expression and virulence and reveals a role for global regulator Hfq. Proc. Natl. Acad. Sci. USA.

[35] Li J., Liu F., Wang Q., Ge P., Woo P.C., Yan J., Zhao Y., Gao G.F., Liu C.H., Liu C. (2014). Genomic and transcriptomic analysis of NDM-1 *Klebsiella pneumoniae* in spaceflight reveal mechanisms underlying environmental adaptability. Sci. Rep..

[36] Crabbé A., Pycke B., Van Houdt R., Monsieurs P., Nickerson C., Leys N., Cornelis P. (2010). Response of *Pseudomonas aeruginosa* PAO1 to low shear modelled microgravity involves AlgU regulation. Environ. Microbiol..

[37] Mastroleo F., Van Houdt R., Leroy B., Benotmane M.A., Janssen A., Mergeay M., Vanhavere F., Hendrickx L., Wattiez R., Leys N. (2009). Experimental design and environmental parameters affect *Rhodospirillum rubrum* S1H response to space flight. ISME J..

[38] Morrison M.D., Fajardo-Cavazos P., Nicholson W.L. (2019). Comparison of *Bacillus subtilis* transcriptome profiles from two separate missions to the International Space Station. NPJ Microgravity.

[39] Wilson J.W., Ott C.M., Quick L., Davis R., Bentrup K.H.Z., Crabbé A., Richter E., Sarker S., Barrila J., Porwollik S. (2008). Media ion composition controls regulatory and virulence response of *Salmonella* in spaceflight. PLoS ONE.

[40] Morrison M.D., Nicholson W.L. (2018). Meta-analysis of data from spaceflight transcriptome experiments does not support the idea of a common bacterial “spaceflight response”. Sci. Rep..

[41] Morrison M.D., Nicholson W.L. (2020). Comparisons of transcriptome profiles from *Bacillus subtilis* cells grown in space versus High Aspect Ratio Vessel (HARV) clinostats reveal a low degree of concordance. Astrobiology.

[42] Kentner D., Sourjik V., Gottesman S., Harwood C.S. (2010). Use of fluorescence microscopy to study intracellular signaling in bacteria. Annual Review of Microbiology.

[43] Guerrero R., Berlanga M. (2007). The hidden side of the prokaryotic cell: Rediscovering the microbial world. Int. Microbiol..

[44] Saier M.H. (2013). Microcompartments and protein machines in prokaryotes. J. Mol. Microbiol. Biotechnol..

[45] Dufrene Y.F., Persat A. (2020). Mechanomicrobiology: How bacteria sense and respond to forces. Nat. Rev. Microbiol..

[46] Persat A. (2017). Bacterial mechanotransduction. Curr. Opin. Microbiol..

[47] Rudner D.Z., Losick R. (2010). Protein subcellular localization in bacteria. Cold Spring Harbor Perspect. Biol..

[48] Wickstead B., Gull K. (2011). The evolution of the cytoskeleton. J. Cell Biol..

[49] Ghosal D., Löwe J. (2015). Collaborative protein filaments. EMBO J..

[50] Wagstaff J., Löwe J. (2018). Prokaryotic cytoskeletons: Protein filaments organizing small cells. Nat. Rev. Microbiol..

[51] Celler K., Koning R.I., Koster A.J., van Wezel G.P. (2013). Multidimensional view of the bacterial cytoskeleton. J. Bacteriol..

[52] Schaechter M. (2015). A brief history of bacterial growth physiology. Front. Microbiol..

[53] Booth I.R. (2014). Bacterial mechanosensitive channels: Progress towards an understanding of their roles in cell physiology. Curr. Opin. Microbiol..

[54] Chan J.M., Guttenplan S.B., Kearns D.B. (2014). Defects in the flagellar motor increase synthesis of poly-gamma-glutamate in *Bacillus subtilis*. J. Bacteriol..

[55] Cairns L.S., Marlow V.L., Bissett E., Ostrowski A., Stanley-Wall N.R. (2013). A mechanical signal transmitted by the flagellum controls signalling in *Bacillus subtilis*. Mol. Microbiol..

[56] Egan A.J.F., Errington J., Vollmer W. (2020). Regulation of peptidoglycan synthesis and remodelling. Nat. Rev. Microbiol..

[57] Thanbichler M., Shapiro L. (2006). Chromosome organization and segregation in bacteria. J. Struct. Biol..

[58] Wang X.D., Rudner D.Z. (2014). Spatial organization of bacterial chromosomes. Curr. Opin. Microbiol..

[59] Le T.B.K., Imakaev M.V., Mirny L.A., Laub M.T. (2013). High-resolution mapping of the spatial organization of a bacterial chromosome. Science.

[60] Lioy V.S., Cournac A., Marbouty M., Duigou S., Mozziconacci J., Espéli O., Boccard F., Koszul R. (2018). Multiscale structuring of the *E. coli* chromosome by nucleoid-associated and condensin proteins. Cell.

[61] Marbouty M., Le Gall A., Cattoni D.I., Cournac A., Koh A., Fiche J.B., Mozziconacci J., Murray H., Koszul R., Nollmann M. (2015). Condensin- and replication-mediated bacterial chromosome folding and origin condensation revealed by Hi-C and super-resolution imaging. Mol. Cell.

[62] Wang X.D., Le T.B.K., Lajoie B.R., Dekker J., Laub M.T., Rudner D.Z. (2015). Condensin promotes the juxtaposition of DNA flanking its loading site in *Bacillus subtilis*. Genes Dev..

[63] Wang X.D., Brandao H.B., Le T.B.K., Laub M.T., Rudner D.Z. (2017). *Bacillus subtilis* SMC complexes juxtapose chromosome arms as they travel from origin to terminus. Science.

[64] Benza V.G., Bassetti B., Dorfman K.D., Scolari V.F., Bromek K., Cicuta P., Lagomarsino M.C. (2012). Physical descriptions of the bacterial nucleoid at large scales, and their biological implications. Rep. Prog. Phys..

[65] Dorman C.J. (2019). DNA supercoiling and transcription in bacteria: A two-way street. BMC Mol. Cell Biol..

[66] Dorman C.J., Dorman M.J. (2016). DNA supercoiling is a fundamental regulatory principle in the control of bacterial gene expression. Biophys. Rev..

[67] de la Campa A.G., Ferrándiz M.J., Martín-Galiano A.J., García M.T., Tirado-Vélez J.M. (2017). The transcriptome of *Streptococcus pneumoniae* induced by local and global changes in supercoiling. Front. Microbiol..

[68] Le T.B.K., Laub M.T. (2016). Transcription rate and transcript length drive formation of chromosomal interaction domain boundaries. EMBO J..

[69] Atack J.M., Tan A., Bakaletz L.O., Jennings M.P., Seib K.L. (2018). Phasevarions of bacterial pathogens: Methylomics sheds new light on old enemies. Trends Microbiol..

[70] Sanchez-Romero M.A., Cota I., Casadesus J. (2015). DNA methylation in bacteria: From the methyl group to the methylome. Curr. Opin. Microbiol..

[71] Sanchez-Romero M.A., Casadesús J. (2020). The bacterial epigenome. Nat. Rev. Microbiol..

[72] Gonzalez D., Kozdon J.B., McAdams H.H., Shapiro L., Collier J. (2014). The functions of DNA methylation by CcrM in *Caulobacter crescentus*: A global approach. Nucleic Acids Res..

[73] Cota I., Bunk B., Spröer C., Overmann J., König C., Casadesús J. (2016). OxyR-dependent formation of DNA methylation patterns in OpvABOFF and OpvABON cell lineages of *Salmonella enterica*. Nucleic Acids Res..

[74] Camacho E.M., Casadesús J. (2005). Regulation of *traJ* transcription in the *Salmonella* virulence plasmid by strand-specific DNA adenine hemimethylation. Mol. Microbiol..

[75] Fioravanti A., Fumeaux C., Mohapatra S.S., Bompard C., Brilli M., Frandi A., Castric V., Villeret V., Viollier P.H., Biondi E.G. (2013). DNA binding of the cell cycle transcriptional regulator *gcrA* depends on N6-adenosine methylation in *Caulobacter crescentus* and other Alphaproteobacteria. PLoS Genet..

[76] Broadbent S.E., Davies M.R., van der Woude M.W. (2010). Phase variation controls expression of *Salmonella* lipopolysaccharide modification genes by a DNA methylation-dependent mechanism. Mol. Microbiol..

[77] Nou X., Skinner B., Braaten B., Blyn L., Hirsch D., Low D. (1993). Regulation of pyelonephritis-associated pili phase-variation in *Escherichia coli*: Binding of the PapI and the Lrp regulatory proteins is controlled by DNA methylation. Mol. Microbiol..

[78] Sánchez-Romero M.A., Busby S.J., Dyer N.P., Ott S., Millard A.D., Grainger D.C. (2010). Dynamic distribution of SeqA protein across the chromosome of *Escherichia coli* K-12. MBio.

[79] Waldminghaus T., Weigel C., Skarstad K. (2012). Replication fork movement and methylation govern SeqA binding to the *Escherichia coli* chromosome. Nucleic Acids Res..

